# Transarterial Embolization-Assisted Necrosis of a Facial Tumor

**DOI:** 10.7759/cureus.29119

**Published:** 2022-09-13

**Authors:** Matthew Dietz, Sachin Jain, Shane Monnett, Amy Deipolyi

**Affiliations:** 1 Surgery, West Virginia School of Osteopathic Medicine, Charleston, USA; 2 General Surgery, Charleston Area Medical Center/West Virginia University, Charleston, USA; 3 Interventional and Diagnostic Radiology, Charleston Area Medical Center/West Virginia University, Charleston, USA

**Keywords:** inoperable head and neck cancer, tumor hemorrhage, tumor necrosis, head and neck squamous cell carcinoma (hnscc), transarterial embolization

## Abstract

The treatment and prognosis of non-operable high-risk head and neck squamous cell carcinoma (SCC) are poor. There is no definitive model for therapy in these cases to date, but strategies that have been utilized include radiation therapy (RT) with or without chemotherapy. Here, we report the effectiveness of arterial embolization with subsequent chemoradiation with cisplatin in a case of advanced oropharyngeal SCC. These interventions resulted in a remarkable tumor burden reduction of a stage IV SCC of the head and neck that had been deemed nonresectable.

## Introduction

Squamous cell carcinoma (SCC) is the most common malignancy to arise in the head and neck region (oral cavity, pharynx, larynx), with a growing incidence worldwide [1]. Data collected from 2012 to 2018 by the Surveillance, Epidemiology, and End Results (SEER) registry reports relative survival at five years to be 86%, 69%, and 40% for local, regional, and distant staged oral cavity and pharynx SCC, respectively [2]. The mainstay treatment of low-stage cases is surgical excision and/or radiotherapy, depending on the location of cancer in the head and neck. Unfortunately, most cases present in later-stage disease, which can preclude the surgical option in the multimodal management of patients [1,2].

The National Comprehensive Cancer Network (NCCN) guidelines include cisplatin chemotherapy with adjuvant radiotherapy (RT) for inoperable high-risk cases of SCC, though the data supporting this approach is questionable [3]. Arterial embolization has not yet been documented as an adequate alternative to surgical excision in non-operable cases of SCC.

Tumor embolization involves the injection of agents to reduce tumor vascularity either via direct puncture or by endovascular techniques. Historically, tumor embolization has been utilized to devascularize lesions with the intention to decrease blood loss, operating time, risk to adjacent tissue and promote tumor necrosis [4,5]. Utilization of this technique has been performed on various tumors as a primary treatment in non-operable circumstances or prior to surgical excision [5]. However, there are no randomized controlled trials supporting the use of endovascular embolization in oropharyngeal SCC to date.

This case analyzes the magnitude of angioembolization-assisted necrosis followed by radiotherapy and cisplatin for a non-operable, high-risk head and neck SCC.

## Case presentation

A 45-year-old man with no known past medical or surgical history presented to the emergency department (ED) complaining of a facial abscess for six months after starting to use intravenous (IV) methamphetamine and heroin in the right neck. Suspecting an abscess, he was initially treated with antibiotics resulting in decreased size of the mass. Despite antibiotics, he continued to have generalized fatigue and fevers associated with severe right-sided facial pain and excessive saliva production. He had no dysphagia or shortness of breath. His social history was significant for smoking two packs per day since he was seven years of age, occasional marijuana use, IV methamphetamines, and heroin use.

**Figure 1:**
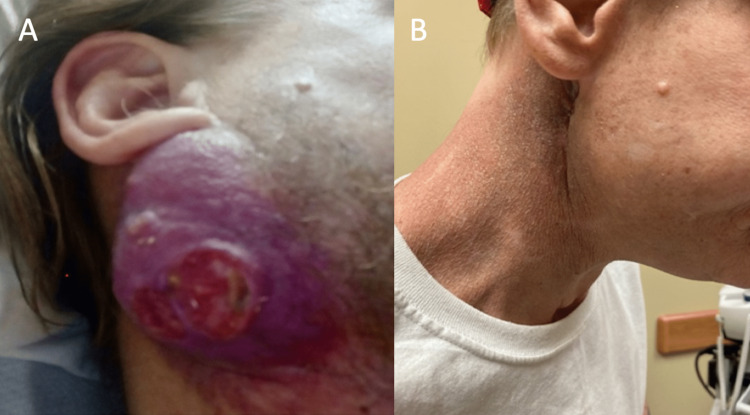
The right neck/facial mass, 05/2021 (A); SCC tumor site with significant reduction of tumor burden along the right mandible, 08/2021 (B) SCC - squamous cell carcinoma

**Figure 2:**
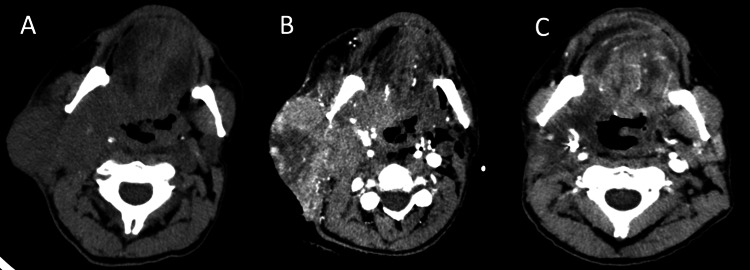
CT of the neck without contrast, 05/2021 (A); CT of the neck with contrast, 06/2021 (B); CT of the neck with contrast after embolization with chemoradiotherapy, 11/2021 (C)

On physical exam, the patient had a mass located at the angle of the right mandible extending to the parotid gland measuring about 6x4 cm with 2 cm of central ulceration (Figure 1A). There was soft tissue swelling to the right buccal mucosa without obvious evidence of penetration of the mucosal layer, and the oropharynx was unremarkable. The patient's vitals were normal on the initial evaluation. The patient's labs were notable for white blood cells (WBC) of 20.6, and he was started on cefepime and vancomycin for concern of sepsis. A CT scan without contrast of the head and neck revealed a large mass of the right superior neck with an extension to the skull base. It was unclear if this was inflammatory or neoplastic in origin. The mass extended into the deep soft tissues abutting the superior larynx, oropharynx, and hypopharynx (Figure 2A). There was an enlargement of submandibular and supraclavicular lymph nodes as well.

Wound cultures were positive for methicillin-resistant Staphylococcus aureus (MRSA), and over the subsequent two weeks, multiple trials of antibiotics were attempted, including cefepime, vancomycin, doxycycline, daptomycin, and ertapenem, without resolution of the patient's sepsis. Ears, nose, throat (ENT) was ultimately consulted, and a parotid biopsy was performed, which was positive for non-keratinizing p16-positive squamous cell carcinoma. Pathology was negative for CK7 but positive for p40 and CK5/6, which is consistent with primary oropharyngeal non-keratinizing squamous cell carcinoma. Clinical staging of the tumor was consistent with T4cN1M1 stage IV disease. The parotid mass was presumed to be a metastatic lesion; however, this was difficult to confirm given the lack of a triple endoscopy exam to directly visualize the oropharynx. In addition, given the evidence of positive nodal disease and advanced metastatic disease, ENT recommended against surgical intervention as clear margins were not attainable, and surgery would require the sacrifice of the facial nerve. The patient was recommended to have RT and cisplatin chemotherapy with palliative consultation, given a poor prognosis. Oncology, radiation oncology, and surgical teams arranged outpatient services for the patient after discharge against medical advice.

Several weeks later, surgery was consulted for the placement of a feeding tube for dysphagia before the initiation of chemoradiation. At that clinic visit, the patient was noted to have a WBC of 42.9, a heart rate of 130 bpm, and a temperature of 38.2° C. He was immediately referred to the ED, where he complained of worsening dysphagia with both solids and liquids. He also had worsening drainage of purulent material from the mass. A repeat contrasted CT of the neck showed that the facial mass had increased in size (Figure 2B). There was a cluster of gas along the posterior inferior margin without any focal fluid collection. The patient was started on vancomycin, cefepime, and metronidazole. ENT was re-consulted and recommended hospice care vs. aggressive resection.

The following day, blood cultures came back positive for Streptococcus, and linezolid was started. A femoral central line was placed, and vasopressor support with norepinephrine was initiated. At this point, the patient was agreeable for gastrostomy tube placement due to progressive weight loss. He was found to have a Mallampati class IV airway. The patient, therefore, consented to an awake tracheostomy placement to protect the airway during the gastrostomy.

**Figure 3:**
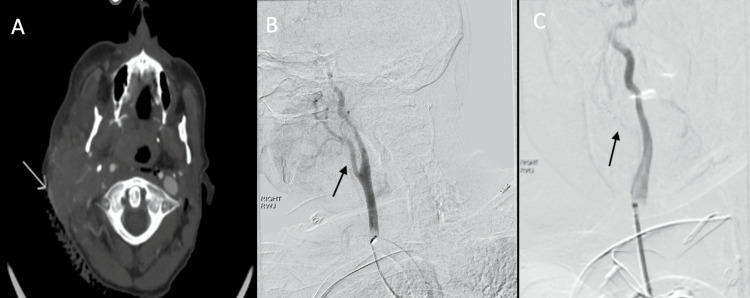
CTA of head/neck with contrast pooling at a superficial aspect of tumor posteriorly (A); initial common carotid digital subtraction image demonstrates that the right external carotid artery supplies a hypervascular tumor (B); after coil embolization of the external carotid, no tumoral enhancement is evident (C, enlargement of image B)

Following the tracheostomy, endoscopic evaluation with the gastroscope revealed no oral cavity or pharyngeal lesions. However, cervical mobilization during gastrostomy tube insertion led to trauma to the neck mass with bleeding that could not be controlled with pressure and hemostatic agents. Vascular surgery was consulted, and a stat CT angiography of the head and neck showed contrast extravasation at the superficial aspect of the right parotid mass (Figure 3A). Following evaluation by vascular surgery, an emergent right external carotid artery coil embolization was completed (Figure 3B-C).

Over the following days, the patient denied any post-embolization symptoms, including stroke or vision loss. His leukocytosis continued to improve, bleeding was well controlled, and he was able to begin radiation therapy. He also had a port placed and was started on weekly treatments of cisplatin. RT was delivered using volumetric-modulated arc therapy (VMAT). The patient completed four cycles of therapy. The first was a prescription dose of 2000 cGY in 10 fractions, followed by 1800 cGY in nine fractions, 600 cGY in three fractions, and 2600 cGY in 13 fractions. The patient began cisplatin 40 mg/at week three, but he only completed two weeks of treatment and refused remaining chemotherapy infusions. 

Following the completion of chemoradiation, the patient followed up in the surgery clinic, where he was noted to have a significant response with tumor shrinkage (Figure 1B). He did experience expected chemoradiation side effects, including moderate fatigue, mucositis, xerostomia, mild nausea, and erythema. Following completion of treatment, his tracheostomy was able to be removed two months after placement, and the gastrostomy tube was removed four months after placement. A CT of the neck with contrast was obtained five months post-angioembolization, which revealed a dramatic decrease in the mass diameter and decreased mass effect on the midline structures of the neck (Figure 2C). He continues to follow up with multidisciplinary teams, including ENT, to determine the management of any residual disease.

## Discussion

SCC of the head and neck has historically been treated with surgical intervention or radiotherapy in low-risk cases with excellent success, but in high-risk cases, outcomes become significantly worse. In these circumstances where patients are poor surgical candidates, treatment is focused on radiation plus or minus concomitant chemotherapy (e.g., cisplatin) as per the NCCN guidelines [3]. Outcomes to date with this recommendation are variable, but improved five-year survival rates have been reported when utilizing concomitant chemotherapy, particularly cisplatin [6-8]. Aside from these recommendations, arterial embolization has not been widely studied for use in the treatment of these tumors other than during the acute onset of hemorrhage. This case required the utilization of emergent coil embolization of the external carotid artery to minimize bleeding, but this intervention may have played a role in tumor burden reduction.

When utilizing arterial embolization, there must be an appreciation of the vascular anatomy to avoid unintended intracranial embolization and/or destruction of healthy tissue adjacent to the tumor [4,9]. By principle, one should identify the primary blood supply from major vessels and target smaller branches directly supplying the tumor. After identification, careful consideration of what material to use (coil, microparticles, liquid agents) needs to be made, given the extent of bleeding and the location involved. The obvious risk is unintentional occlusion of the vasculature to healthy tissue leading to ischemia and symptomatic deficits from blockage of branches of the external and internal carotid arteries (pain, facial palsy, paralysis, vision loss, stroke, etc.). Following angioembolization, an interval is allowed for tumor necrosis before pursuing further therapies.

In the review of the literature, there is no study that has thoroughly analyzed long-term outcomes in patients treated with embolization with subsequent radiotherapy plus or minus chemotherapy. Some cases report sole utilization of embolization for hemorrhage control, but none investigated subsequent treatment, including radiation and chemotherapy. However, several institutions have reported patients continuing their designated chemo- or radiotherapy protocol following successful angioembolization. Shetty et al. analyzed the use and efficacy of angioembolization in patients presenting with sudden and massive hemorrhage with unresectable and recurrent metastatic head and neck cancer and found that out of 26 patients, rebleeding occurred in three. Overall, palliative embolization was effective with a bleed-free interval from several weeks to 21 months. This report also mentions that several patients initiated palliative chemotherapy following successful embolization, but no other details were given [10].

Rzewnicki et al. identified that selective embolization for treatment of hemorrhage out of 76 patients with inoperable head and neck cancer resulted in control of bleeding in 86% of cases. Of those cases where bleeding resolved, all patients then continued their designated chemotherapy regimens or Co-60 radiotherapy. However, long-term follow-up and overall patient outcomes following the completion of each patient's designated therapy were not provided in this report [11].

The most detailed report looking at outcomes involving embolization came from Sesterhenn et al. The authors analyzed seven cases of high-risk head and neck cancer which presented with tumor hemorrhage and received emergent embolization measures to control bleeding. Of these cases, six were SCC (two larynxes, two oro-hypopharynx, one hypopharynx, and one oropharynx). Three cases utilized coils to occlude the external carotid artery, with one case also occluding the facial artery to control bleeding. The median survival for these three cases was 23 weeks. Among the other three SCC cases, polyvinyl-alcohol particles (PVA) were used to occlude the facial and maxillary artery, superior thyroid artery, and lingual artery. Survival for these cases was 168, 19, and 12 weeks respectively. Of all six cases, none were a primary disease, and all had received radiotherapy plus or minus chemotherapy before embolization [12].

The lack of literature investigating outcomes after embolization is likely due to the rarity that a patient with non-operable advanced head and neck cancer presents with sudden hemorrhage as well as the limited number of facilities with the expertise to manage this situation. Of the cases available, it is difficult to assess survival outcomes as the majority focus on bleed-free outcomes vs. patient prognosis with continued palliative care. The gap in existing literature makes it difficult to compare and assess the magnitude of tumor reduction solely from embolization compared to chemoradiation intervention.

Advanced inoperable SCC of the head and neck entails poor overall survival (OS) due to locoregional recurrence of disease or distant metastasis. The more advanced the stage of the disease typically correlates with the poorer OS. Several reports have attempted to identify more specific prognostic factors, including human papillomavirus (HPV), p16, E6, and E7 status, tobacco use, and nodal status, to guide therapy options in patients with advanced head and neck cancer. In primary oropharyngeal SCC, it seems that patients are treated similarly, irrespective of tumor marker status, but p16-positive patients have been shown to have better outcomes regardless of HPV positivity [13]. Tobacco use has also been shown to minimize the RT effects by diminishing positive outcomes associated with HPV-positive tumors. Ang et al. suggest a significant inverse relationship between increasing pack years and overall survival [14].

Despite this information, our case identifies a p16-positive T4 oropharyngeal tumor, which manifested in a man with a 38-year two-pack per day smoking and IV drug use history that responded remarkably to arterial embolization followed by seven weeks of total 70 Gy of VMAT radiation with two total cisplatin doses. On most recent imaging, a five-month post-embolization CT scan of the neck with contrast showed a significant decrease in the size of tumor bulk along the suprahyoid, parapharyngeal, and oropharyngeal regions. Compared to prior imaging, there was also decreased infiltrative involvement of the right lateral oropharyngeal wall and sublingual space with decreased nodular thickening of the right aryepiglottic folds and epiglottis, and there was a noted decrease in size of the right submandibular lymph node.

It should be expected that, given this patient's history and diagnosis, he will require continuous follow-up to assure there is no locoregional recurrence or metastatic disease. Given the dramatic response of nodal disease and the presumed metastatic parotid lesion to treatment, the patient may benefit from triple endoscopy at this point to attempt to identify a primary lesion. Head and neck cancer tends to reappear within two years following treatment [15]. With the involvement of embolization, in this case, it is difficult to project subsequent recurrence, overall survival, or subsequent comorbidities associated with treatment.

To investigate the effectiveness of radiotherapy, we reviewed outcomes in cases of palliative radiotherapy in similarly staged head and neck cancer. Iqbal et al. reviewed 24 studies that investigated outcomes in cases that had similarly staged head and neck cancer and received palliative radiotherapy. From this report, overall survival ranged from three to 17 months, with most reporting an average survival of six months. These cases all utilized different RT protocols, with most delivering hypofractionated amounts, but the authors suggest the response is dose-related [16]. Of these 24 studies, Stevens et al. differentiated SCC from other histopathologic tumors showing a median OS of under six months amongst 112 patients while using variable radiotherapy protocols up to 70 Gy over 35 fractions [17]. This could support our patient's unconventional response to embolization with chemoradiotherapy as he is currently 11 and 13 months post-chemoradiotherapy and embolization intervention, respectively. However, this report did not separate OS by different stages (II-IVC) or location (pharynx, nasopharynx, other) of SCC. It did show that histopathological diagnosis other than SCC resulted in improved survival (hazard ratio of 0.52, p<0.01) [17].

While investigating palliative chemoradiotherapy versus solo therapy, most studies and organizations, including the NCCN, recommend combined therapy depending on the intention of maximizing symptomatic relief vs. improved survival with an increased potential for toxicity. As an example, Kumar et al. revealed an increase in median OS of four months amongst patients who received chemoradiotherapy compared to solely RT [18]. In patients who had at least partial response after four weeks were provided additional chemo- or RT, while others were not subjected to further treatment to prevent unwanted toxicities in exchange for further treatment resistance. This study is in accordance with the larger phase III clinical trials performed, which highlight improved median OS with chemoradiotherapy (CRT) vs. radiotherapy alone [19]. This is important to consider as every patient has the potential to respond differently; however, the best potential for improved OS continues to be with combined therapy regimens.

In summary, this case of high-risk head and neck cancer was successfully treated with a combination of arterial embolization with chemoradiation, suggesting the possibility of embolization as an adjunct intervention to improve outcomes.

## Conclusions

Embolization is a successful intervention to treat acute hemorrhage in patients with advanced head and neck cancer. Embolization may additionally render inoperable advanced head and neck cancer more susceptible to subsequent chemoradiotherapy. Larger studies are required to explore the significance of embolization-assisted tumor necrosis in combination therapy intervention. Additionally, our report supports the use of combined therapy protocols in palliative care to provide improved OS and outcomes in patients with advanced head and neck cancers.
